# The Role of Basal Ganglia and Its Neuronal Connections in the Development of Stuttering: A Review Article

**DOI:** 10.7759/cureus.28653

**Published:** 2022-08-31

**Authors:** Deepa G, Shrikrishna B H, Ujwal Gajbe, Brij Raj Singh, Anupama Sawal, Trupti Balwir

**Affiliations:** 1 Anatomy, Datta Meghe Medical College, Datta Meghe Institute of Medical Sciences, Nagpur, IND; 2 Otolaryngology-Head and Neck Surgery, All India Institute of Medical Sciences, Nagpur, Nagpur, IND; 3 Anatomy, Jawaharlal Nehru Medical College, Datta Meghe Institute of Medical Sciences, Wardha, IND

**Keywords:** white matter, substantia nigra, stuttering, gray matter, dysfluency, basal ganglia

## Abstract

Dysfluent speech has the potential to lower one's standard of living drastically. Although there is a lot of theoretical support for basal ganglia dysfunction in developmental stuttering, there isn't any imaging data to back it up. According to several studies, there is a difference in gray matter volume between people who stammer and those who don't. According to studies, the right inferior longitudinal fasciculus and the uncinate fasciculus have higher fractional anisotropy (FA) than fluent controls. A high fractional anisotropy means good white matter integrity in these areas. In children who stutter, grey matter volume was higher in the Rolandic operculum, middle frontal gyrus, superior temporal gyrus, and inferior parietal lobule. These regions are found to be more active in adults who stammer as their speech fluency improves. Stuttering is previously linked to structural deficiencies in the corpus callosum. However, there are differences in the directionality of the findings between studies, which are unknown. According to current theories, stuttering is caused by a breakdown in the integration of auditory data in speech motor planning, which affects behavior tasks that rely on basal ganglia structures. According to some studies, connectivity in the left inferior frontal gyrus (IFG) and basal ganglia of persons with stuttering (PWS) was significantly reduced. Still, it was more robust in the left supplementary motor cortex (SMC) and premotor cortex (PMC) (primary motor cortex). In the Broca's region, there was also decreased perfusion and spectroscopic indicators of neuronal density. Spontaneous speech is more affected by stuttering than conversation, reading, sentence repetition, or singing. As per the dual process theory of language formation, the basal ganglia are essential for formulaic phrases, but the left hemisphere is important for innovative, freshly constructed sentences. According to current theories on their functional traits and connections to cortical areas of control, the basal ganglia are the complex networks in charge of organizing, initiating, carrying out, and controlling motor behaviors.

Given the distinct neuroanatomical characteristics of people who stutter, more research into this cohort is required to further our understanding of the illness. The primary goal of this review article is to fill in any knowledge voids between the neuroanatomical structure of the basal ganglia and the onset of stuttering.

## Introduction and background

Dysfluent speech is the defining characteristic of developmental stuttering. It is theorized that the brain systems in charge of controlling and producing speech are implicated in the etiology of stuttering. Children's stuttering is thought to be caused by predetermined neuroanatomical or neurophysiological variances, and it's connected to abnormalities throughout the entire brain network that controls speech. The quality of life can be greatly reduced by developmental stuttering. The basal ganglia (BG) dysfunction in developmental stuttering has a lot of theoretical support, but there isn't any imaging evidence to support it. Only a few speech-related cortical regions, including the precentral gyrus, anterior, middle frontal gyrus, inferior frontal gyrus, and superior temporal gyrus, exhibit abnormalities in adults who stutter. Adults who stutter differ from control participants who speak fluently in terms of speech-related neuroanatomical structures and underlying white matter networks. However, it is yet unknown whether these variants develop early in the onset of stuttering or if they do so as a result of events that occur during life. Children who stutter had noticeable alterations in Gray Matter Volume (GMV) compared to children who are recovered stutterers and fluent speakers. 

To better understand the condition, more research on this group is required, given the distinctive neuroanatomical characteristics found in people who stutter. Our review article's main goal is to understand the pathophysiology of this illness and to fill up any knowledge gaps between the neuroanatomical structure of the BG and the onset of stuttering.

## Review

Method

MEDLINE/PubMed and PubMed Central were the primary electronic databases, with Medical Subject Headings (MeSH) keywords included. "Basal ganglia" and "Stuttering" were the two MeSH terms combined. Our research includes studies on children and adults (ages 6 to 44) written in English and published within the last ten years. Gender differences were not considered. This review excluded studies that used animals or were written in a language other than English. Our study set out to identify neuroanatomical anomalies in the BG as a distinct stuttering risk factor. All authors of this review read the information found in the papers' titles, abstracts, and free full-text versions. The entire text, abstract, and Digital Object Identifier (DOI) information for these publications, the title of the publication, the name of the journal, the date of publication, and the DOI, were manually entered on Microsoft Excel sheets. The team members re-examined each item on the chart to highlight points in another chart that backed our research goal. There was no Research and Ethics Committee approval because this was a typical literature review.

Result

Fifteen papers altogether were retrieved for this evaluation of the literature. These 15 articles were all full-text, open-access articles as given in Table 1.

**Table 1:** MeSH terms were used to locate relevant studies for the review. MeSH, Medical Subject Headings

MeSH keyword searches for 'Basal Ganglia' and 'Stuttering'	The number of records
Total number of records	122
Following the application of inclusion and exclusion criteria	
Published in the last ten years	72
Literature in full text for free	43
Humans	22
Age 6-44 years	15

Discussion

The defining feature of developmental stuttering, which affects 8% of children and 1% of the general population, is dysfluent speech. Unintentional repetitions, prolongations, and blocking of sounds, syllables, or words are characteristics of the neurogenic speech disorder known as stuttering. The brain systems responsible for speech production and control are hypothesized to be involved in the etiology of stuttering. Stuttering in children is assumed to result from preset neuroanatomical or neurophysiological differences and is linked to anomalies across the entire brain network responsible for producing speech [1]. Stuttering during development can significantly lower the quality of life. Although there is a lot of theoretical support for BG dysfunction in developmental stuttering, there isn't any imaging data to back it up. Only a few speech-related cortical areas that show abnormalities in adults who stutter are the precentral gyrus, anterior, middle frontal gyrus, inferior frontal gyrus, and superior temporal gyrus [2]. The area under the inferior frontal gyrus in adults with stuttering exhibits decreased frontal lobe activity on functional Magnetic Resonance Imaging [3]. According to the research above, adults who stutter have distinct speech-related neuroanatomical structures and underlying white matter networks than control participants who speak fluently. It is still unclear, nevertheless, whether these variations arise earlier in the initiation of stuttering or if they do so as a result of lifelong events. Chang et al. reported in a study of children aged 9 to 12 years who stutter, former stutterers, and able speakers that youngsters who stammer have noticeable changes in Gray Matter Volume (GMV) compared to children who are recovered stutterers and fluent speakers [4]. According to Chang et al., in kids who stammer, the right inferior longitudinal fasciculus and uncinate fasciculus have higher fractional anisotropy (FA) than fluent controls.

In contrast to fluent controls, the Beal et al. study described neuroanatomic anomalies in stuttering children's gray and white matter [1]. The findings suggest that the Rolandic operculum and the right inferior frontal gyrus are essential regulators of persistent developmental stuttering. Stuttering children have lower GMV in the right and left inferior frontal gyri than fluent controls. The inferior frontal gyri are critical regions in the brain network that control motor control of speech. Lower GMV in the inferior frontal gyri is associated with stuttering early in development. Stutterers may have unstable speech motor programs due to this underdevelopment, as evidenced by their wildly inconsistent speech movements [5]. In a study of school-aged children who have been stuttering for more than two years, Beal et al. discovered reduced grey matter volume in the inferior frontal gyrus's left pars orbitalis. This finding supports the Chang et al. study of GMV in stammering children [4]. GMV was higher in the superior temporal gyrus, middle frontal gyrus, inferior parietal lobule, and Rolandic operculum of children who stutter. These regions are also more active during fluency-enhancing speaking situations in adults who stammer [6].

In children who stutter, less white matter volume in the corpus callosum's forceps is minor compared to controls who speak fluently may indicate a lack of brain resources for interhemispheric communication [1]. The corpus callosum's structural deficiencies have previously been linked to stuttering. However, there are differences in the findings' directionality among research, and it is unknown why. Adults who stutter have higher levels of White Matter Volume (WMV) in networks underlying the superior temporal gyrus, insula, right inferior frontal gyrus and precentral gyrus, and corpus callosum [7]. Beal et al.'s research, however, found no WMV anomalies in kids who stammer outside of the corpus callosum. Beal et al. utilized an 8mm smoothing filter, which was wide enough to blur findings across the pars orbitalis, pars triangularis, and pars opercularis anatomical boundaries. As a result, they found it challenging to accurately determine which of these areas is less than the other. 

While research on GMV variations in the BG between people who stammer and control persons has found inconsistent findings, Montag et al. claim that the ventral striatum is a crucial neurological mechanism behind developmental stuttering [8]. The Diffeomorphic Anatomical Registration using Exponentiated Lie algebra (DARTEL) method was used in their study's utilization of a more significant sample and analysis of the available brain data [8]. According to them, those who stutter have more gray matter in their right ventral BG. According to Rosenfield, neurological disorders are thought to cause idiopathic or developmental stuttering. The condition significantly impacts the motor and auditory parts of the left hemisphere and the fiber tracts that connect them [9]. According to one theory, stuttering is brought on by a breakdown in the integration of auditory feedback in speech motor planning. This affects how well people do behavioral tasks that need the BG structures [10].

Dopamine, a crucial BG neurotransmitter, has been linked to stuttering in studies [11]. In several investigations, the basal ganglia's structural characteristics are different in stutterers [8]. Most MRI studies on stuttering are underpowered statistically and with insufficient sample sizes, according to Sowman et al. [12]. According to their study, the reduction in gray matter volume in the striatum is seen in children who stutter and can also be seen in adults who stutter. This finding adds weight to evidence that nominates the area as playing a causal role in stuttering [12]. According to Shen and Sterr, each group would require a minimum of 25 individuals to provide significant results [13]. This supports Sowman et al.'s viewpoint. 

Parallel loops make up the BG circuits, which use thalamic projections to channel input from cortical regions back to the cortex [14]. Smooth and fluid movements are facilitated by the BG circuitry, which may explain why patients who stutter have trouble executing smooth motor sequences [15,16]. The right inferior premotor cortices, the insula, the cerebellum, and an under-activation of the auditory cortices were all overactive in the first meta-analysis on stuttering that used activation likelihood estimation (ALE) approaches [6].

Although the role of the BG in stuttering has received extensive research, the exact location has been debated, and the results of meta-analyses have not yet been encouraging. We are assured that we will be able to provide convincing evidence of right ventral striatum neurostructural alterations [8]. According to Neef et al., adults who stutter exhibit anatomical changes in the right ventral striatum [17]. They examined how the motivational and social modulation of stuttering might be explained by reward-related features of speech production [18]. 

In Magnetic Resonance Imaging (MRI) investigations, the number of false positive results is inversely proportional to the sample size. Small sample sets and outdated, less precise processing pipelines were used in most earlier investigations on the BG and stuttering [19]. Montag et al. discovered that those who stutter have more significant putamen regions in their brains and that this difference is especially noticeable in the right hemisphere [8]. The correctness of inter-subject alignment and an exact match of brain anatomy in common space determine the validity of the morphometric analysis.

Tissue segmentation, denoising techniques, and DARTEL normalization were all part of the processing pipeline [8]. Most earlier investigations into the BG and stuttering used modest sample sets and dated inaccurate processing methods. The scientists discovered that the right putamen region had a greater volume than the left putamen region in a study involving 74 people. The authors of the study propose that increased GMVs in the right ventral striatum in stutterers could be explained as a brain process of compensation that may be useful to offset speech issues throughout a person's lifetime. The study had significant drawbacks, though, including the inability to correlate stuttering severity with ventral striatal regions and the failure to collect complete SSI-III scores from a sizable proportion of stutterers. The author concluded that it is unclear how anatomical alterations in the BG impact the pathogenesis and etiology of stuttering. However, the ventral striatum appears to be crucial.

Qiao et al. collected data from functional magnetic resonance imaging at rest from 44 stutterers and 50 typically developing fluent speakers [20]. They looked for networks of stable, functionally linked brain areas using Independent Component Analysis and Hierarchical Partner Matching to see if connectivity varied significantly across diagnostic groups. According to studies, adult stutterers have white matter volume in the middle temporal gyrus, inferior frontal gyrus, corpus callosum, and superior temporal gyrus than those who don't. They also have a lower volume of gray matter in the putamen, caudate, and inferior frontal gyrus.

According to functional MRI studies, the left Inferior Frontal Cortex in Broca's area (IFG), the corticostriatal-thalamo-cortical (CSTC) loops, and the BG may all be dysfunctional in stuttering, and this dysfunction may extend to language sequencing in a domain-specific way [20]. It is possible to find potential biomarkers for psychological illnesses using machine learning techniques [21-23]. The authors used machine learning to evaluate the efficacy of Granger Causality and Independent Component Analysis (ICA) functional connectivity measures in distinguishing typically developing (TD) fluent speaker controls from PWS. The authors proposed that they would find changes in brain connectivity in PWS in Broca's region and the accompanying language loop, as well as within the cortical-subcortical neural circuits that enable human speech, based on previous designs and stuttering research in practice. According to Qiao et al., PWS's functional connectivity in the left inferior frontal gyrus and BG was significantly lower than in the primary motor cortex and left SMA. In the Broca's region, there was also decreased perfusion and spectroscopic indicators of neuronal density. These findings suggest that PWS patients have faulty language circuits that aid speech preparation and motor sequences. Brain areas that varied between stuttering groups were found using machine learning techniques, and these differences were linked to improved classification accuracy. 

Speech disfluency in PWS could be due to a reduced innate functional network in the IFG [20]. Both the cortical and subcortical motor areas' inner phonatory loop, which regulates speech production, and the outer linguistic cortical loop, which aids speech-language control and auditory self-monitoring, may be dysfunctional in stuttering [24,25]. The Qiao et al. study had several limitations, including a wide age range of participants, the use of self-report measures of stuttering severity, and the potential impact of concomitant conditions in the five stuttering participants. It is critical to confirm these findings using various methods, including EEG.

Natural vocal activities, including repeating, reading, singing, and conversational speech include motor speech [26]. It was traditionally believed that everyday speech's articulatory and vocal qualities were constant throughout tasks and that dysarthria always occurred regardless of the demands of the activity. Singing is one vocal technique linked to neurobiological disparities in performance. Following damage to or removal of the left hemisphere, singing is retained, and listeners are more likely to understand singing than spontaneous speech [27,28]. According to studies, innovative, propositional language is stored and processed differently than learnt, routinized utterances.

Additionally, individuals with undamaged BG exhibit normal or enhanced levels of repeated speech and formulaic expressions [29]. Recited speech uses internal models that have been remembered, whereas conversational speech uses newly formed internal models of motor function. Repetition and reading make use of externally supplied models. In comparison measurements of speech rate in patients with Parkinsonism, whose rates varied by task, Van Lancker Sidtis et al. found that the conversational dysfluency greater than in other activities is consistent with these findings [29]. The increased dysfluency in recited and spontaneous speech is most likely due to improved linguistic planning. The researchers studied a person with Parkinson's disease who had severe motor speech impairment when other speech tasks were triggered but had severe dysfluency in spontaneous speech. Stuttering impacted automatic speech more than conversation, reading, sentence repetition, or singing. According to the dual process theory of language formation, the BG are essential for formulaic phrases, but the left hemisphere is important for innovative, freshly constructed sentences. The basal ganglia are thought to be complex networks responsible for the planning, beginning, controlling, and monitoring of motor behaviors, according to current views on their functional characteristics and their link to cortical areas of control. According to the authors' findings for singing, stuttering is a task-specific illness because oral-motor dysfunction only manifests itself during talking and not chewing, singing, or humming [30]. Why singing has such a special meaning in neurological diseases is still unknown. According to Van Lancker Sidtis et al., motor speech performance is most stressed during a spontaneous speech, while speech task influences motor speech competence [29].

Additionally, the results support the idea that Karaoke practice could help a trained or novice vocalist with neurogenic acquired stammering. Using a computational model, Civier et al. investigated potential neural causes of stuttering. They explain two stuttering-related findings: white matter fiber anatomical abnormalities beneath the left precentral gyrus and evidence of significantly higher dopamine levels in the dorsal striatum [31]. According to the author, failure to inhibit the previous step and to activate the subsequent stage are specific instances of either of the two most common descriptions of any insistent occurrence. Stuttering is most clearly defined by sound/syllable repetitions disregarding segmental (phonemic) boundaries. Both pathways are occupied by the basal ganglia, which is also involved in the supplementary motor region and may be involved in the condition. Acquired stuttering has been linked to basal ganglia lesions following strokes and traumatic brain injuries [32]. The striatum of the BG has the densest dopamine innervation in the brain, and studies have shown that D2R (dopamine receptor D2) blockers can effectively reduce stuttering. However, D2R antagonist therapy has adverse side effects [33].

According to functional MRI studies, the globus pallidus may be involved in stuttering, but It is difficult to determine which BG nuclei are causing the activation changes. The premotor cortex (PMC) is likely involved in stuttering because it codes for syllables, sends projections to the BG, and receives projections from the thalamus. To address issues caused by dysfluencies, the researchers used an expanded version of the Gradient Order Directions Into Velocities of Articulators (GODIVA) model that considers both cortical and subcortical areas. The model can predict the entire arrangement of blood-oxygenation-level-dependent (BOLD) responses seen through the simulated brain areas. The GODIVA model replicates brain cell activity and explains how the brain can store random utterances that follow a speaker's grammar [34]. According to the GODIVA hypothesis, the SSM choice cells, which correspond to the thalamic cells that code for the sounds "go" and "di," interact with the cortical column that codes for those sounds. The GODIVA model uses the lateral inhibition the basolateral gyrus offers, which enables the brain to make choices more quickly if the requirements for releasing a plan are met.

Additionally, the putamen cells' feedforward lateral inhibition is used by the BG to boost contrast enhancement. Each motor command sent to the motoneurons is copied to the putamen by the motor cortex, which projects to the brainstem through corticostriatal fibers. As a result, the putamen can predict when the current syllable will end and swiftly go on to the next. Each motor command sent to the motoneurons is copied to the putamen by the motor cortex, which projects to the brainstem through corticostriatal fibers. As a result, the putamen can predict when the current syllable will end and can swiftly go on to the next. Blocks and prolongations might be understood using the extended GODIVA model as instances where the motor program for the subsequent syllable was not timely activated. The scientists reasoned that one of these anomalies would result from the other during brain development and predicted that stuttering would have high dopamine levels and white matter damage.

Cler et al. examined the brains of people who stutter and a group of people who are generally fluent and matched for age and gender using a multi-parameter mapping methodology. They discovered that stutterers had lower basal ganglia iron concentrations than fluent people [35]. People who stutter had more gray matter, higher mean values in the left putamen and frontal lobe, higher mean values in the left caudate nucleus, and more gray matter in both the right and left hemispheres, according to their findings. Increased dopamine levels may contribute to increased iron levels, which may explain why people who stutter have higher iron concentrations in their gray matter. Parkinson's disease, which is also connected to stuttering, may have elevated iron levels. Similar results from a recent study using ultrasonography to identify higher iron deposition in the substantia nigra in stutterers were also reported [36].

Metzger et al. looked at the whole-brain functional network to see how basal ganglia regions harmonize and rearrange sensorimotor brain nexus in people who stutter. They discovered that the substantia nigra activity was related to the severity of stuttering and that thalamus and globus pallidus were more active [37]. The primary basal ganglia substrates of dopamine production are the substantia nigra pars compacta (SNc) and substantia nigra pars reticularis (SNr). In chronic developmental stuttering, the substantia nigra is a central hub that orchestrates and reorganizes sensory brain networks. The researchers used an fMRI (functional magnetic resonance imaging) paradigm that consistently produces activity in this region. They wanted to know if the coordination of cortico-striatonigral pathways causes stuttering. The substantia nigra (SN) psychophysiological interaction analysis, which employs a correlation study of physiological time-varying signal change, was used to accomplish this. Their study found a link between severe stuttering and increased SN activity during response anticipation. This finding supports the hypothesized hyperdopaminergic stuttering state. The single-nucleotide polymorphisms (SNPs) of Parkinson's disease patients have a lower late copy number variants (CNV) amplitude. This demonstrates that the SNc is involved in planning a motor response in stuttering. It has been discovered that SN activity and stuttering severity are correlated. Still, this result is difficult to interpret because the SN is divided into two functionally separate areas: dopaminergic SNc neurons control striatal activity, and GABAergic SNr neurons curb nuclei of the thalamus. 

Task-related functional magnetic resonance imaging (fMRI) studies have shown several brain regions linked to PDS (persistent developmental stuttering). A measure of neural synchronization called resting state functional connectivity shows the relationships between spontaneous blood oxygen level-dependent (BOLD) fluctuations (RSFC). Stuttering is caused by an improper connection between the brain's temporal gyrus and pre-supplemental motor area (SMA) and the basal ganglia.

Yang et al. examined adults who stutter for basal ganglia connections to the cerebellum and thalamocortical networks [38]. The authors found a positive link between the right inferior frontal gyrus and the right lobule VI and a negative association between the vermis III and the left cingulate gyrus and stuttering severity. The findings of this investigation show that PDS alters intrinsic interconnections in cerebellar networks, supporting earlier findings that the cerebellum plays a significant role in PDS.

The left temporal-striatal tract connected the frontal and temporal lobe cortex in PWS compared to Person with No Stuttering (PWNS). At the same time, Cieslak et al.'s diffusion spectrum imaging (DSI) investigation indicated that the left and right arcuate tracts were altered in PWS compared to PWNS [39]. Seven out of eight PWS individuals in their study lacked streamlines in the arcuate fasciculus. This suggests that PWS and PWNS have different structural characteristics. Chang and Zhu used diffusion tensor imaging (DTI)-based probabilistic tractography to investigate children who stammer. They discovered decreased structural connectivity between the middle frontal gyrus and the left superior temporal gyrus, as well as between the left pars opercularis and the middle temporal gyrus [25]. 

The frequency of stuttering was positively related to speech-related basal ganglia activation using positron emission tomography (PET) scan and fMRI [40]. The basal ganglia are a group of well-connected nuclei that play a role in intentional action selection and execution, promoting voluntary movement while inhibiting unwanted or disruptive movement and managing non-motor behaviors [41].

Neurological dysfunction in the left-sided cortico-basal ganglia-cortical network has been linked to stroke-induced neurogenic stuttering, according to Theys et al. [42]. In a study by Saltuklaroglu et al., persons with stuttering produced Mu spectra with reduced beta amplitudes across conditions, suggesting reduced forward modeling capacity [43]. According to previous fMRI research, several behavioral tasks that evaluate various elements of timing and temporal processing activate an overlapping network of areas, including the cerebellum, basal ganglia (BG), SMA, prefrontal, and parietal cortices. For example, somatosensory, motor, auditory, and visual time are all supported by brain regions interacting with the BG and SMA, which have been considered to form a core timing network [44]. The summary of the reviewed articles can be seen in Table 2.

**Table 2:** Summary of Reviewed Articles

Reference	Study Design	Year of Publication	Sample Size (n)	Finding
Beal et al. [1]	Case-control study	2013	22	The right inferior frontal gyrus and the Rolandic operculum are crucial regulators of persistent developmental stammering. In children that stammer, there is also less grey matter volume in the left putamen and bilateral inferior frontal gyri.
Montag et al. [8]	Case-control study	2019	70	More grey matter is present in the right ventral basal ganglia of subjects who stammer.
Sowman et al. [12]	Case-control study	2017	54	Adult stutterers exhibit the same decrease in striatal grey matter volume as children who stutter. This discovery strengthens the case for the region's causal involvement in stuttering.
Qiao et al. [20]	Case-control study	2017	94	Stuttering may be caused by dysfunction in the basal ganglia, the left IFG (Inferior Frontal Cortex in Broca's area), and the Corticostriatal-thalamo-Cortical (CSTC) loops. This disruption may also affect language sequencing in a domain-specific manner.
Chang et al. [25]	Case-control study	2013	56	Children who stutter may have distinct basal ganglia-thalamocortical and auditory-motor network development, which may impact the speech planning and execution procedures required to attain fluent speech motor control.
Van Lancker Sidtis et al. [29]	Descriptive study	2012	1	Dysfluency in conversations is higher than in other pursuits. It is most likely because of better linguistic preparation that there is more dysfluency in both recited and spontaneous speech.
Cler et al. [35]	Case-control study	2021	73	Basal ganglia iron concentrations were lower in stutterers than in fluent individuals. More grey matter, higher mean values in the left putamen and frontal lobe, higher mean values in the left caudate nucleus, and more grey matter in both the right and left hemispheres were all present in individuals who stutter.
Metzger et al. [37]	Case-control study	2018	27	The severity of stuttering and increased activity in the thalamus and globus pallidus are correlated with substantia nigra activity. The substantia nigra is a key hub that coordinates and reorganises sensory brain networks in chronic developmental stuttering.
Yang et al. [38]	Case-control study	2016	34	The vermis III and the left cingulate gyrus are negatively correlated with stuttering severity, while the right inferior frontal gyrus and the right lobule VI are positively correlated.
Cieslak et al. [39]	Case-control study	2015	16	When compared to people who don't stammer, the left and right arcuate tracts are different in stutterers.
Connally et al. [40]	Case-control study	2018	33	Using PET and fMRI, it was discovered that stuttering frequency was positively correlated with speech-related basal ganglia activation.
Colato et al. [41]	Case-control study	2021	1288	Regional patterns of grey matter volume loss are identified by spatial independent component analysis (ICA), some of which are relevant to contemporaneous disability and some of which indicate future development.
Theys et al. [42]	Case-control study	2013	37	The cortico-basal ganglia-cortical network on the left side has been related to neurological malfunction that causes neurogenic stuttering after stroke.
Saltuklaroglu et al. [43]	Case-control study	2017	54	People who stammer had Mu spectra with smaller beta amplitudes under all situations, which would indicate a lower forward modelling capacity.
Chang et al. [44]	Case-control study	2016	40	The lack of a correlation between rhythm network connectivity and rhythm discrimination in children who stammer may be a significant contributing element to the aetiology of stuttering. Children who stutter have poorer rhythm network connectivity.

Beal et al., Qiao et al., Cler et al. and Yang et al. concur on the role of inferior frontal gyri in the development of stuttering. Beal et al. and Cler et al. propose a disturbance in the GMV in the left putamen. However, the GMV was less in the former and more in the latter. Disturbance in the neuronal network of basal ganglia was proposed by Montag et al., Qiao et al., Chang et al., Cler et al., Connally et al., and Theys et al. Lower iron concentration in basal ganglia in stutterers was proposed by Cler et al. Increased neuronal activity in thalamus and globus pallidus was proposed by Metzger et al. Difference in the anatomy of the arcuate tracts in stutterers was proposed by Cieslak et al.

Strengths and limitations

The majority of the papers in this evaluation were clinical trials and observational studies. There was no meta-analysis included. Another drawback of this study is the exclusion of non-English literature and the use of a small electronic database (PubMed and PubMed Central). The original content of this article, which was created more than ten years ago, contained specific components that were lifted from other sources, such as the definition.

## Conclusions

The quality of life is greatly reduced by developmental stuttering. The Rolandic operculum and right inferior frontal gyrus are essential in developing persistent stuttering. Studies comparing the basal ganglia's GMV between stutterers and controls have produced varying results. Adult stutterers exhibit the same decrease in striatal GMV as children who stutter. This discovery strengthens the case for the region's causal involvement in stuttering. According to studies, dopamine, a crucial basal ganglia neurotransmitter, has been linked to stuttering. People who stutter may have trouble developing fluent, smooth motor sequences because the basal ganglia circuitry facilitates such motions. According to functional MRI studies, stuttering is caused by a malfunction in the left inferior frontal cortex in Broca's area, basal ganglia, and corticostriatal-thalamo-cortical loops, which extends to language sequencing in a domain-specific way. The severity of stuttering and increased activity in the thalamus and globus pallidus are correlated with substantia nigra activity. The substantia nigra is a key hub that coordinates and reorganizes sensory brain networks in chronic developmental stuttering. Children who stutter may have distinct basal ganglia-thalamocortical and auditory-motor network development, which may impact the speech planning and execution procedures required to attain fluent speech motor control. Basal ganglia iron concentrations were lower in stutterers than in fluent individuals in some studies. More gray matter, higher mean values in the left putamen and frontal lobe, higher mean values in the left caudate nucleus, and more gray matter in both the right and left hemispheres were all present in individuals who stutter. Using PET and fMRI, it was discovered that stuttering frequency was positively correlated with speech-related basal ganglia activation. The cortico-basal ganglia-cortical network on the left side has been related to a neurological malfunction that causes neurogenic stuttering after a stroke. It will take a more extensive investigation in the future to confirm the findings above.
